# The effects of levosimendan and dobutamine in experimental bupivacaine-induced cardiotoxicity

**DOI:** 10.1186/1471-2253-13-28

**Published:** 2013-10-03

**Authors:** Ulku Kandemir, Fikret Maltepe, Baran Ugurlu, Necati Gokmen, Asli Celik

**Affiliations:** 1 Mus State Hospital, Clinic of Anaesthesia and Reanimation, (Formerly Dokuz Eylul University, Department of Anaesthesia and Reanimation, Izmir, Turkey), Mus, Turkey; 2 Department of Anaesthesia and Reanimation, Dokuz Eylul University, Medical Faculty, Izmir, Turkey; 3 Department of Cardiovasculary Surgery, Dokuz Eylul University, Medical Faculty, Izmir, Turkey; 4 Department of Experimental Laboratory Animal Science, Dokuz Eylul University, Medical Faculty, Izmir, Turkey

**Keywords:** Bupivacaine, Cardiotoxicity, Levosimendan, Dobutamine, Rat

## Abstract

**Background:**

Accidental intravenous exposure to bupivacaine is highly cardiotoxic and may lead to death. Positive inotropic agents are usually utilized in resuscitative efforts. We have compared the efficacy of levosimendan, a novel inotropic agent, with dobutamine and their combination in a rat model of bupivacaine intoxication.

**Methods:**

Twenty-eight male Wistar albino rats weighing between 250-300 g were divided into these four groups: control (C), levosimendan (L), dobutamine (D) and dobutamine+levosimendan (D+L). Bupivacaine was administered at a dose of 3 mg/kg/min until cardiac arrest occurred or for 120 min. ECG, heart rate, blood pressure, arterial blood gases, and end tidal CO_2_ levels were monitored. Levosimendan was administered as a bolus of 12 μg/kg for 10 min and continued as an infusion at 0.3 μg/kg/min. Dobutamine was infused at a dose of 3 μg/kg/min. The time required for a 50% and 75% decrease in heart rate and blood pressure with a total time to cardiac arrest and bupivacaine dose for obtaining cardiac arrest were analyzed.

**Results:**

Time periods for heart rate reductions of 50% and 75% were significantly longer in groups L (903, 1198 s), D (984, 1542 s) and L+D (1705, 3152 s) compared with the control group (345, 538 s p < 0.001). Median times to mean blood pressure reductions of 50% and 75% were 399 - 504 s in the control group, 1005 -1204 s in group L, 685 - 1009 s in group D and 1544- 2982 s in group L+D, and the difference was significant compared with the control group. Median time duration to asystole was 703 s in the control group compared with 1385 s in group L, 1789 s in group D and 3557 s in group L+D. Time to cardiac arrest was significantly higher in all 3 study groups. It was also significantly higher in the L+D group compared with both groups L and D separately.

**Conclusion:**

A combination of dobutamine with levosimendan significantly increased survival times in this bupivacaine-induced toxicity rat model compared with the control, levosimendan, and dobutamine groups.

## Background

Bupivacaine is one of the most cardiotoxic local anesthetic agents. Despite its known adverse effects on the heart, it is widely used by anesthesiologists and surgeons for localized procedures and nerve blocks [1]. Although it is usually a safe agent that can be used without any adverse effects at recommended doses, there are reports in the literature that accidental intravenous administration of bupivacaine during epidural or peripheral nerve blocks do exist and lead to cardiac arrest, which is refractory to resuscitation in some patients [2].

Intravenous administration of local anesthetics and particularly bupivacaine results in reduced cardiac contractility arrhythmias and hypotension [3], which can lead to ventricular tachycardia, fibrillation, asystole, and complete heart block in humans [4]. Resuscitation after bupivacaine-induced cardiovascular collapse usually involves the use of sympathomimetic agents to restore coronary circulation, and adrenaline, isoproterenol, dopamine, dobutamine, and amrinone, a phosphodiesterase inhibitor, have been evaluated in animal models with variable results [4]. Current guidelines recommend the utilization of intralipid solutions in cases of cardiac arrest following local anesthetic toxicity with bupivacaine [3,5]. However, the underlying mechanisms of this novel therapy have not been fully understood. Therefore, it is necessary to search for new drugs or drug combinations that could be useful for the treatment of this life-threatening medical condition.

Levosimendan is a calcium sensitizer, which binds to cardiac troponin C and increases the calcium sensitivity of myocytes. Levosimendan increases contractility of the cardiac muscle without increasing intracellular calcium [6]. Levosimendan is currently indicated for the treatment of chronic heart failure and has been proven beneficial in certain acute cardiotoxic events [7-10].

Therefore, we have hypothesized that levosimendan may help resuscitative efforts after accidental exposure to intravenous bupivacaine. To determine its possible use following cardiovascular collapse after bupivacaine-induced cardiotoxicity, we have evaluated the effects of levosimendan and dobutamine treatment in an animal model of acute bupivacaine toxicity. The primary outcome variable was duration to cardiac arrest.

## Methods

This study was approved by our institutional review board for animal studies (Dokuz Eylul University School of Medicine (DEUSM) Local Animal Experiments Ethical Committee 24.12.2010, The number of protocol: 75-2010, Yilmaz O), and the experiments were performed at the Dokuz Eylül University multidisciplinary animal laboratory. In all, 28 male Wistar albino rats weighing between 250-300 g were used. Rats were anesthetized with an intraperitoneal injection of Uretan® (Sigma Chemical Company, St. Louis, MO, USA) at a dose of 1 g/kg. The animals were placed in a supine position with tapes, and a tracheostomy was performed with a 16-G venous cannula. Two 20-G venous cannulae were placed in both tail veins, and 0.9% saline was administered at a bolus dose of a 0.5 ml and continued as an infusion at 2 ml/h. A 26-G catheter was placed in the right carotid artery for arterial pressure monitoring; a flush solution containing 100 U/ml of heparin was used for this purpose. Vecuronium (Norcuron®, Organon, Netherlands) was administered at a dose of 0.03 mg/kg, and the animals were connected to a small animal ventilator (Kent Scientific Pressure Controlled Ventilator, Norfolk, CT, USA). The ventilator was set at room air with a tidal volume of 8 ml/kg and rate of 55/min. The end tidal carbon dioxide level was kept between 25-30 mm Hg (Anesthetic Gas Monitor Type 1380, Brüel&Kjaer, Denmark). The animals were kept at 37°C. Electrocardiogram was monitored thru needle leads placed in all four extremities, and the ECG and blood pressure waveforms were recorded with a Biopac mp 35 polygraph recording system (Biopac System, Inc., Santa Barbara, CA, USA).

After a 15-min stabilization period, baseline heart rate, systolic, diastolic and mean arterial pressure, QRS duration and arterial blood oxygen saturation, pH, and carbon dioxide levels were recorded. An intravenous infusion of bupivacaine (Marcaine heavy, Astra Zeneca Istanbul, Turkey) at a 3 mg/kg/min dose was initiated after the stabilization period and continued until either cardiac arrest occurred or an infusion duration of 120 min was reached. Elapsed time from the start of bupivacaine infusion to cardiac arrest was defined as the major endpoint of the study.

Study animals were divided into the following four treatment groups: control, levosimendan, dobutamine, and dobutamine+levosimendan groups. Infusion of the study drugs was initiated at the first sign of cardiotoxicity, which was determined as a 20% increase in the duration of the QRS complex.

Control group (n = 7), Group C: No additional medication was given; only a saline infusion was maintained.

Levosimendan group (n = 7), Group L: At the first sign of cardiotoxicity, levosimendan (Simdax® Abbott, USA) was started from the second tail vein catheter at a bolus dose of 12 μg/kg for 10 min, and the infusion was continued at 0.3 μg/kg/min until cardiac arrest.

Dobutamine group (n = 7), Group D: At the first sign of cardiotoxicity, dobutamine (Eczacibasi-Baxter, Turkey) at a dose of 3 μg/kg/min was started and continued until cardiac arrest.

Dobutamine+Levosimendan group (n = 7), Group D+L: At the first sign of cardiotoxicity, levosimendan was given as a bolus of 12 μg/kg for 10 min and was followed by an infusion of 0.3 μg/kg/min. A dobutamine infusion was simultaneously started at a dose of 3 μg/kg/min and continued until cardiac arrest.

Blood pressure, ECG, and heart rates of the subjects were recorded continuously. Time durations for 50% and 75% decreases in mean blood pressure and heart rate as well as the total time to cardiac arrest were recorded.

### Power analysis

The primary outcome of this study was the time that elapsed from the start of bupivacaine infusion until asystole. In a previous report comparing saline and clonidine pretreatment in the prevention of bupivacaine-induced cardiotoxicity in rats, the investigators used the same primary outcome that was used in this study [9]. In this study, asystole time was determined 546 ± 82 s in the bupivacaine+saline group [9]. Using this data and the detection of a 20% change in the asystole time with an α error of 0.05 and a power of 80%, it was calculated that the sample size should be at least seven rats per group. For 2 by 2 analysis when p value is taken as 0.01 the study power is calculated less than 80%.

### Statistical analysis

The results are presented as medians because the number of subjects was too small to allow a normal distribution. Group comparisons determining homogeneity were made using the Kruskall-Wallis test, significance was accepted at a p value less than 0.05. The results were analyzed in a binary fashion with study groups compared with the control group using the Mann-Whitney U test and significance was calculated at a p value less than 0.0083 with Bonferroni correction. The study data were analyzed using version 11.5 of SPSS software.

## Results

The baseline variables of the subjects in all four groups were similar. There was no difference in relation to weight, heart rate, blood pressure, QRS width, arterial blood pH, pO_2_ and pCO_2_ values (Table 1).

**Table 1:** Baseline values of the study subjects are given as median and minimum and maximum

**Groups**	**Control**	**Levosimendan**	**Dobutamine**	**Dobutamine+Levosimendan**
Weight (g)	286 (260-313)	219 (268-336)	292 (269-338)	295 (255-315)
Heart rate/minute	348 (238-427)	349 (309-400)	381 (230-456)	343 (233-481)
Systolic blood pressure (mmHg)	113 (105-129)	122 (105-131)	112 (94-150)	117 (96-134)
Diastolic blood pressure (mmHg)	67 (52-86)	78 (54-86)	72 (48-100)	65 (45-87)
Mean blood pressure (mmHg)	82 (70-100)	93 (71-101)	81 (63-116)	84 (67-97)
QRS width (s)	0.023 (0.022-0.025)	0.022 (0.019-0.026)	0.022 (0.019-0.026)	0.023 (0.020-0.026)
Arterial blood pH	7.33 (7.14-7.39)	7.31 (7.23-7.42)	7.36 (7.21-7.38)	7.34 (7.24-7.39)
Arterial blood pO_2_ (mmHg)	182 (118-216)	148 (105-219)	153 (128-288)	149 (118-288)
Arterial bood pCO_2_ (mmHg)	30 (21-39)	29 (28-38)	30 (26-38)	33 (24-42)

Comparison between four groups showed no statistically significant difference.

The infusion time to first sign of cardiotoxicity, which was a 20% increase in the QRS duration, was statistically similar in all four groups. Hemodynamic changes, arrest times, and total lethal dose of bupivacaine are presented in Table 2.

**Table 2:** Results of the control group with the 3 study groups

**Study groups**	**Control**	**Levosimendan**	**Dobutamine**	**Dobutamine+Levosimendan**
Initial QRS > 20% (s)	162 (101-342)	173 (103-403)	153 (105-410)	159 (90-432)
Initial arrhythmia (s)	203 (129-549)	455 (153-850)	448 (249-1367)	310 (97-567)
Heart rate < 50% (s)	345 (214-583)	903* (301-1848)	984 *(672-2814)	1705 †* (1039-3009)
Heart rate < %75 (s)	538 (353-961)	1198* (358-7014)	1542 * (895-4092	3152 †* (1747-6519
Mean arterial pressure < %50 (s)	399 (245-665)	1005* (243-1592)	685* (514-1992)	1544 †* (874-3003)
Mean arterial pressure < %75 (s)	504 (381-814)	1204* (298-6028)	1009* (698-4013)	2982 †* (1542-6897)
Cardiac arrest time (s)	703 (403-1049)	1385* (412-6552)	1789 * (944-4742)	3357 †* (1825-7200)
Total Bupivacaine dose (μg)	8.48 (5.03-13.11)	17.62 * (5.15-90.0)	22.36 * (11.80-59.20)	41.90 †* (22.80-90.0)

Data is given as median with minimum and maximum values.

*p < 0.05 compared to the control group, *†p < 0.008* for pairwise comparison of all 4 groups.

The median times to a 50% and 75% decrease in heart rate were significantly longer in groups L (903-1198s), group D (984, 1542 s) and L+D (1705, 3152 s) compared with the control group (345, 538 s p < 0.05). There was also no significant difference between groups D and L+D but the study was under powered for pairwise analysis.

Median time duration for a decrease in mean blood pressure of 50% and 75% was also longer in all three study groups compared with the control group (399, 504 s). It was longest in the L+D group [1544, 2982 s, followed by group L (1005, 1204 s) and group D (685, 1009 s)]. While comparison between group L+D and group L and group D reached significance, the comparison between group D and group L did not reach significance but the study was underpowered in this regard.

Three subjects in the B+L group did not develop cardiac arrest during the 120-min study duration; all other subjects eventually developed asystole. The median time duration to asystole was 703 s in the control group compared with 1385 s in group L, 1789 s in group D and 3557 s in group L+D. Time to cardiac arrest was significantly higher in all three study groups. It was also significantly higher in the L+D group compared separately with both groups L and D. The total infused dose of bupivacaine showed a similar pattern in all three groups that was significantly higher than in the control group, and also was higher in the L+D group compared with groups L and D.

## Discussion

The results of this study have demonstrated that both levosimendan and dobutamine prolong the time for cardiac arrest development after bupivacaine-induced cardiotoxicity.

Deleterious effects of accidental intravenous administration of bupivacaine leading to severe cardiovascular collapse and shock are well documented [11-13]. Various agents have been evaluated for alleviating the cardiotoxic effects of bupivacaine in both animal studies and human case series. Experimental treatment protocols have included amrinone, isoprotrenol, insulin, clonidine, adrenalin, and phenylephrine. Currently only dobutamine and lipid infusion are generally recommended [14-24]. Despite all resuscitative efforts with dobutamine and lipid infusion treatment, accidental intravenous bupivacaine exposure can be fatal, and newer treatment options may prove helpful [3].

Levosimendan has proven beneficial effects on cardiac dysfunction following cardiac surgery, myocardial infarction, cardiac resuscitation, or sepsis [25-31]. Despite its growing use in cardiac failure patients, levosimendan was evaluated in only two previous animal studies for treating experimental cardiotoxicity caused by local anesthetic agents. In one study with ropivacaine, a levosimendan infusion caused a marked improvement in heart rate, as well as generated pressure and cardiac output, compared with a normal saline infusion in isolated pig hearts [32]. These promising results led to another study involving anesthetized pigs that evaluated the effect of levosimendan on bupivacaine-induced cardiotoxicity [33]. In this randomized study, 20 pigs received a bupivacaine infusion at a dose of 2 mg/kg/min until a 55% decrease in mean arterial pressure. The study group received 80 μg/kg levosimendan for 10 min followed by an infusion of 0.7 μg/kg/min. Although cardiac output, ejection fraction, and stroke power/end-diastolic volume measurements showed rapid improvement with levosimendan, there was no time-group effect difference in overall recovery and mortality. Heart rate was significantly higher in the levosimendan group, and this was interpreted as a possible sign of improved recovery with levosimendan [33].

Encouraging results from the isolated pig heart and pig animal studies have led us to design our study using a rat model. In the present study, in contrast to the previous study, we continued the bupivacaine infusion until cardiac arrest. Another difference was that we also compared levosimendan infusion with dobutamine and a dobutamine+levosimendan combination treatment. The reasoning being that in clinical situations with severe cardiotoxic effects, these drugs would most likely be used in combination to revive the patient. A bupivacaine infusion dose of 3 mg/kg/min was chosen because a previous study in rats demonstrated rapid cardiovascular collapse with a similar dose [34].

Our results did show a consistent trend in improved survival times with a levosimendan infusion. In the group that received levosimendan infusion alone, infusion durations leading to a 50% and 75% decrease in both heart rate and mean blood pressure were longer compared with the control group, but the differences did not reach statistical significance. Survival times and the mean bupivacaine dose required for cardiac arrest did show a statistically significant improvement. Dobutamine infusion alone showed a similar effect on blood pressure and heart rate reduction times, and the effect on heart rate showed statistical significance. Survival time and total bupivacaine doses were also significantly higher compared with the control group but showed no difference compared with the levosimendan infusion group.

A combination of levosimendan with dobutamine showed a marked improvement in all study endpoints except time to first arrhythmia. These differences reached statistical significance for time durations for 50% and 75% decreases in both mean blood pressure and heart rate compared with the control group and also compared with the levosimendan and dobutamine groups. Three rats in the dobutamine+levosimendan group reached the 120-min termination point without developing cardiac arrest. The mean time to cardiac arrest and the mean infused dose of bupivacaine was more than fourfold higher compared with the control group, and these parameters were significantly improved compared with the dobutamine and levosimendan groups.

It is logical to assume that a combination of dobutamine and levosimendan would augment their respective inotropic effect because they affect different steps of the cardiac myocyte calcium metabolism [35,36]. Levosimendan also has a vasodilatory effect, but an increase in cardiac output may have compensated for this vasodilatory effect; our results did not show a decrease in blood pressure [37]. This vasodilatory effect may explain why the blood pressure decrease times did not reach statistical significance in the levosimendan alone infusion group. Predictably, heart rate was also unaffected by levosimendan as much as dobutamine.

Our results suggest an improved recovery with levosimendan in bupivacaine-induced cardiotoxicity. Dobutamine does have a similar effect, but this improvement in recovery can be augmented greatly if dobutamine is combined with levosimendan. Our study has shown that dobutamine+levosimendan is safe in this context, i.e., there was no increased arrhythmogenic effect. We believe this combination can administered in patients with acute bupivacaine intoxication and cardiovascular collapse because this drug combination is clinically approved for the management of cardiac failure [26]. Further studies are required to compare the efficacy of levosimendan to lipid treatment for resuscitation following bupivacaine-induced toxicity.

## Conclusions

Our study has shown an increased survival time with both levosimendan and dobutamine infusion following bupivacaine toxicity in rats. Greatest benefit was recorded with administration of dobutamine and levosimendan together with survival times significantly longer compared to all groups. This combination may prove useful in treating circulatory collapse following bupivacaine toxiticity.

## Competing interests

The authors declare that they have no competing interests.

## Authors’ contributions

UK carried out animal experiments, FM conceived the study, BU participated in design of the study and drafted the manuscript, NG participated in the design of the study and contributed to the statistical analysis, AC contributed to the experiments. All authors read and approved the final manuscript.

## Pre-publication history

The pre-publication history for this paper can be accessed here:

http://www.biomedcentral.com/1471-2253/13/28/prepub
